# International Comparison of Surgical Management Practices for Necrotizing Enterocolitis in Neonates: Insights from Cohorts in the Netherlands and Finland

**DOI:** 10.1055/a-2536-4468

**Published:** 2025-03-18

**Authors:** Bineta E. Lahr, Otis C. van Varsseveld, Daphne H. Klerk, Mikko Pakarinen, Antti Koivusalo, Jan B.F. Hulscher

**Affiliations:** 1 Department of Neonatology, University Medical Centre Groningen, Groningen, The Netherlands; 2 Department of Intestinal Diseases, European Reference Network for Inherited and Congenital Anomalies, Rotterdam, Zuid-Holland, The Netherlands; 3 Department of Pediatric Surgery, University Medical Centre Groningen, Groningen, The Netherlands; 4 Department of Pediatric Surgery, University of Helsinki Children's Hospital, Helsinki, Uusimaa, Finland

**Keywords:** preterm infants, necrotizing enterocolitis, surgery, practice variations, neonatology

## Abstract

**Introduction:**

Surgical management of necrotizing enterocolitis (NEC) can result in significant morbidity and mortality. Surgical management varies in the absence of international evidence-based guidelines. We aimed to gain insight into practice variation between expert centers in the Netherlands and Finland.

**Materials and Methods:**

Bicentric retrospective cohort study including all infants treated surgically for NEC (Bell's stage ≥IIA) in two centers in the Netherlands and Finland between 2000 and 2021. Main outcomes were preoperative, intraoperative, and 3-month postoperative characteristics.

**Results:**

We included 191 patients (122 Dutch and 69 Finnish). Median gestational age and birth weight were lower in Finnish patients (median [min.–max.]: 25 + 4/7 [23 + 0/7–39 + 0/7] vs. 28 + 2/7 [23 + 6/7–41 + 6/7], *p* < 0.001, and 795 g [545–4,000] vs. 1,103 g [420–3,065], *p* < 0.001). Indication for surgery was mostly pneumoperitoneum in Finnish patients (56.5% vs. 37.7%; *p* = 0.02) versus clinical deterioration on conservative treatment in Dutch patients (51.6% vs. 23.2%; *p* < 0.001). A fixed-bowel loop was also more often an indication in Finland (20.3% vs. 3.3%; *p* < 0.001. Ostomy creation was more common in Finnish patients (92.8% vs. 53.3%; *p* < 0.001) and primary anastomosis in Dutch patients (29.5% vs. 4.4%; *p* < 0.001). Open-close procedures occurred in 13.9% of Dutch cases, versus 1.4% of Finnish cases ( *p* = 0.004). Mortality at 3 months was comparable when excluding open-close procedures (24.8% vs. 19.1%; *p* = 0.46).

**Conclusions:**

We observed varying populations, indications for surgery, and surgical approaches in NEC between the Netherlands and Finland. The occurrence of open-close procedures is 10-fold higher (13.9% vs. 1.4%) in the Netherlands compared to Finland. Long-term outcomes remain to be studied. These results point toward significant practice variation and strengthen the need for European management guidelines.

## Introduction

Necrotizing enterocolitis (NEC) is a devastating intestinal inflammatory disease and the most common cause of gastrointestinal morbidity and mortality in the neonatal intensive care unit (NICU). 1 2 NEC mainly occurs in preterm and extremely low birth weight infants. In both Finland and the Netherlands, NEC incidence was around 13% in cohorts of extremely preterm infants (<28 weeks of gestational age [GA]) from 2005 to 2013. 3 4 Due to advancements in neonatal care and changes in guidelines for resuscitation of neonates, an increasing population of very and extremely preterm neonates was seen, adding to the incidence of NEC. 3 5

The initial approach to treating NEC involves discontinuing enteral feedings, implementing nasogastric aspiration, administering antibiotics, and providing respiratory and hemodynamic support as needed. 3 Surgical intervention is indicated in the case of pneumoperitoneum, clinical deterioration despite maximal conservative treatment therapy, a fixed bowel loop, or in cases where diagnostic uncertainty necessitates abdominal exploration. 6 7 8 9 10 However, with the exception of pneumoperitoneum, these indications are neither straightforward nor generally accepted. The percentage of patients requiring surgical intervention varies between 27 and 52%. 3 4 11 12 In addition to laparotomy, infants diagnosed with surgical NEC may be treated by primary peritoneal drainage. When compared, there seems to be no difference in rates of death or neurodevelopmental impairment between the two treatments. 13 Despite research efforts over the last decades, infants suffering from NEC still face significant mortality and morbidity, with worse outcomes for those needing surgical management. The mortality rate is around 35% in infants treated surgically for NEC, compared to 21% for those with NEC treated medically. 6 14 Survivors of NEC may experience persistent health issues, including short bowel syndrome in up to 23% and an increased risk of neurodevelopmental delay, particularly following surgical treatment. 1 13 15 16 17

Uncertainties remain about indications for surgery, timing of surgical intervention, and the selection of surgical procedures in patients with NEC. 6 7 18 No internationally established evidence-based guidelines for surgical NEC are currently in place. Therefore, the decision to proceed with surgery or whether comfort care is in the infant's best interest, is complicated for the medical team and parents alike. Even though the decision-making factors are being identified, clinical and therapeutic characteristics determining successful NEC surgery need to be further explored. 19 20 Assessing practice variation in surgical NEC in relation to outcomes may play a pivotal role in optimizing surgical intervention for NEC. International benchmarking initiatives such as the European Reference Network for Inherited and Congenital Anomalies (ERNICA) may aid toward optimization and standardization of future NEC care.

The aim of this retrospective cohort study was to assess the differences in presentation, management, and postoperative characteristics of infants surgically treated for NEC between 2000 and 2021 at the University Medical Center Groningen (UMCG) and Helsinki University Hospital (HUS). We hypothesize that surgical management of NEC varies between countries and centers in the absence of international evidence-based guidelines. Identifying key differences in NEC presentation and surgical management between these two centers may lead to new insights for improvement of care and focused research for this devastating disease.

## Material and Methods

### Patients and Setting

This bicenter, retrospective cohort study was performed in the UMCG and HUS. Both the centers are academic referral centers for surgical NEC with a level IV NICU. Both the centers are formally recognized as Centers of Expertise for NEC and participate as such in ERNICA. For our study, we included infants of all gestational ages surgically treated for NEC between January 1, 2000, and December 31, 2021. Review of patient charts for this study was approved by the institutional review board in both the centers. Informed consent was not required due to the retrospective nature of the study. In accordance with hospital regulations patients may opt out from any research, but there were no opt outs among the studied population.

The diagnosis of NEC (modified Bell's stage ≥IIA) was established through a combination of clinical signs (abdominal distension, bilious aspirates, and anal blood loss) and abdominal X-ray findings including pneumatosis intestinalis and/or portal venous gas, as classified by the modified Bell's staging criteria. 21 22 Abdominal ultrasonography was not part of the standard diagnostic protocol for NEC at either of the participating centers during the study period. Patients were excluded if they had focal/spontaneous perforation or if NEC was related to a congenital heart disease. 23 NEC associated with congenital heart disease is believed to have a distinct pathophysiology, primarily driven by intestinal ischemia, which could have confounded our analysis of practice variations. 23

Indications for NEC surgery throughout the study period comprised: (1) intestinal perforation identified as pneumoperitoneum on abdominal X-rays; (2) radiologically confirmed NEC with deterioration despite maximum conservative treatment and no clinical improvement within 24 hours; or (3) a fixed bowel loop on serial abdominal X-rays (>24 hours). 9 Panintestinal necrosis was defined as extensive bowel necrosis deemed incompatible with a reasonable quality of life by the surgical team, resulting in an open-close procedure. Contraindications for surgery were poor general condition beyond resuscitation, or when the medical team and parents did not deem surgery to be in the best interest of the child. In both the centers, the primary surgeon was always a fully trained general and pediatric surgeon with experience in managing patients with NEC. At the UMCG, at least one laparotomy was performed in the NICU during the study period, whereas at the HUS, the majority of surgeries were conducted in the NICU without transportation to the operating room.

### Data Collection

Clinical data were retrospectively collected from electronic medical records and recorded in a separate, secure, pseudonymized database ( *REDCap 13.7.19* - © 2023 Vanderbilt University). Clinical data was collected by one of the authors (BL, AK) and randomly checked for every 10 patients by a second author (DK, MP). Unclear cases or diagnoses were discussed with one of the senior authors. The following variables were collected: (1) patient information including gestational age at birth, birth weight, sex, head circumference, multiple pregnancy, hemodynamically significant ductus arteriosus (hsPDA), intraventricular hemorrhage (Papile grades I–IV 24 ), respiratory distress syndrome (based on blood gas analyses or radiographic appearances 25 ) mechanical ventilation, other congenital malformations; (2) preoperative data including postnatal day of NEC onset (defined as the first day on which pneumatosis or portal venous gas was visible on abdominal X-ray) and surgery, hours between NEC diagnosis and surgical treatment, maximum Bell's stage, indication for surgery, hematological and biochemical values (CRP, lactate, pH, thrombocytes); (3) intraoperative data including type of primary procedure performed, location of NEC, removal of ileocecal junction; (4) postoperative data at 3 months including mortality, days of postoperative mechanical ventilation, recurrent NEC, post-NEC stenosis (clinically relevant bowel strictures, not at a former anastomotic site, with radiological and/or surgical confirmation, after the acute phase of NEC), 26 short bowel syndrome (parenteral nutrition >60 days postoperatively 27 28 ), relaparotomy (excluding stoma closure), days of postoperative parenteral feeding, and conjugated bilirubin levels. Stoma refeeding was not a routine practice at either center.

### Statistical Analysis

We used Statistical Package for the Social Sciences (SPSS) version 28.0 (Windows) software for the statistical analysis. For all descriptive data, normally distributed data were reported as mean ± standard deviation (SD) and non-normally distributed data were reported as median with their minimum and maximum value (min.–max.). Categorical variables were reported as frequency and percentage. The distribution was analyzed using histograms and Q-Q-plots. To assess the differences in continuous variables between the two independent groups we used the T-test in parametric distributions and the Mann-Whitney U test in nonparametric distributions. For categorical variables Fisher's exact test was used. We performed a univariable logistic regression analysis to evaluate the association between the time period of NEC diagnosis and mortality, excluding open-close procedures. All tests were conducted two-sided and a *p* -value <0.05 was considered statistically significant.

## Results

Between January 1, 2000 and December 31, 2021, 389 UMCG patients and 90 HUS patients were diagnosed with NEC modified Bell's stage ≥IIA. Data of 122 surgically treated NEC patients from the UMCG and 69 from the HUS were included. Patient demographics are shown in Table 1 . Gestational age was significantly lower in HUS patients, as was birth weight. Other demographic characteristics did not differ significantly between the two centers. There were four patients with congenital malformations in the UMCG group, including choanal atresia, cheilognathopalatoschisis, SRY-positive 46, XX testicular disorder of sexual development, and hypospadia.

**Table 1:** Demographic and clinical data

	UMCG ( *n* = 122)	HUS ( *n* = 69)	*P* -value
Gestational age (weeks + days)	28 + 2/7 (23 + 6/7–41 + 6/7)	25 + 4/7 (23 + 0/7–39 + 0/7)	** <0.001 a**
Birth weight (g)	1,103 (545–4,000)	795 (420–3,065)	** <0.001 a**
Male	71 (58.2)	45 (65.2)	0.36 b
Head circumference (cm)	25 (22–35)	23.5 (21–33)	0.17 a
Multiple pregnancy	30 (24.6)	12 (17.4)	0.28 b
First	9 (30.0)	3 (25.0)	
Second	20 (66.7)	9 (75.0)	
Third	1 (3.3)	0 (0.0)	
hsPDA	48 (39.3)	32 (46.4)	0.36 b
Intraventricular hemorrhage	31 (25.4)	24 (34.8)	0.19 b
Grades I–II	28 (90.3)	18 (75.0)	0.60 b
Grades III–IV	3 (9.7)	6 (25.0)	** 0.07 b**
Mechanical ventilation	112 (91.8)	65 (94.2)	0.77 b
Other congenital malformations	4 (3.3)	0 (0)	0.30 b

Abbreviations: hsPDA, hemodynamically significant patent ductus arteriosus based on echocardiographic confirmation; HUS, Helsinki University Hospital; UMCG, University Medical Center Groningen.

Notes: Data are presented as median (Min.–Max.) or frequency and percentage.

*P* -values of less than 0.05 (two-tailed) were considered statistically significant.

a Mann-Whitney U test.

b Fisher's exact test.

### NEC Characteristics

The median postnatal day of NEC onset was 9 days in both the hospitals, and median postnatal day of surgery was 11 (2–77) and 10 (1–95) in the UMCG and HUS, respectively ( Table 2 ). The proportion of patients receiving surgery within 24 hours after NEC diagnosis was significantly higher in the HUS group compared to the UMCG group (30/69, 43.5% vs. 31/122, 25.4%; *p* = 0.04). In the majority of HUS cases, surgical intervention was instigated by pneumoperitoneum (39/69, 51.6% vs. 46/122, 37.7%; *p* = 0.02). For the UMCG group, surgery was indicated most often by deterioration despite maximum conservative treatment (63/122, 51.6% vs. 16/69, 23.2%; *p* < 0.001). Fixed bowel loop for more than 24 hours was more prevalent indication for surgery in HUS compared to UMCG (14/69, 20.3% vs. 4/122, 3.3%; *p* < 0.001). Median gestational age of HUS patients was 3 weeks lower among those with pneumoperitoneum (25 + 1/7 [23 + 1/7–37 + 6] vs. 28 + 0/7 [24 + 5/7–39 + 4/7]; *p* < 0.001), and 1 week lower in those with deterioration despite maximum conservative treatment as an indication for surgery (27 + 0/7 [23 + 1/7–39 + 0/7] vs. 28 + 0/7 [23 + 6/7–41 + 6/7]; *p* = 0.04). There were no significant differences in preoperative CRP, lactate, pH, or thrombocytes between the UMCG and HUS groups.

**Table 2:** Preoperative NEC characteristics

	UMCG ( *n* = 122)	HUS ( *n* = 69)	*P* -value
Postnatal day of NEC onset (days)	9 (1–66)	9 (0–62)	0.81 a
Postnatal day of surgery (days)	11 (2–77)	10 (1–95)	0.44 a
Hours between NEC diagnosis and surgical treatment b	48 (1–504)	24 (5–576)	0.53 a
< 24 h	31 (27.9)	30 (43.5)	**0.04** c
24–72 h	57 (51.4)	26 (37.7)	**0.09** c
> 72 h	23 (20.7)	13 (18.8)	0.85 c
Maximum Bell's stage (IIIA or IIIB)	107 (87.7)	50 (72.5)	**0.02** c
Indication for surgery			
Pneumoperitoneum * Gestational age (weeks + days)*	47 (38.5) 28 + 0/7 (24 + 5/7–39 + 4/7)	39 (56.6) 25 + 1/7 (23 + 1/7–37 + 6)	**0.02** c **<0.001** c
Deterioration despite maximum conservative treatment * Gestational age (weeks + days)*	63 (51.6) 28 + 0/7 (23 + 6/7–41 + 6/7)	16 (23.2) 27 + 0/7 (23 + 1/7–39 + 0/7)	**<0.001** c **0.04** c
Fixed bowel loop * Gestational age (weeks + days)*	4 (3.3) 29 + 1/7 (26 + 5/7–32 + 5/7)	14 (20.3) 26 + 4/7 (23 + 0/7–35 + 5/7)	**<0.001** c 0.08
Explorative surgery * Gestational age (weeks + days)*	8 (6.6) 31 + 5/7 (25 + 6/7–40 + 4/7)	0 (0.0) *N/A*	0.05 c
Lab values preoperative			
CRP (mg/L)	80 (0.3–422)	58 (3–311)	0.18 a
Lactate (blood gas)	2.3 (0.7–14.7)	2.6 (1.0–18.0)	0.24 a
pH	7.29 (6.90–7.48)	7.24 (6.98–7.55)	0.06 a
Thrombocytes (10 ^9^ /L)	134 (10–751)	125 (27–424)	0.77 a

Abbreviations: CRP, C-reactive protein; HUS, Helsinki University Hospital; NEC; necrotizing enterocolitis; UMCG, University Medical Center Groningen.

Notes: Data are presented as median (Min.–Max.) or frequency and percentage.

*P* -values of less than 0.05 (two-tailed) were considered statistically significant.

a Mann-Whitney U test.

b Missing data: 11 UMCG, 0 HUS. Not counted toward denominator of percentage.

c Fisher's exact test.

### Surgical Characteristics

Resection with ostomy was significantly more prevalent as the primary surgical procedure in HUS patients compared to UMCG patients (64/69, 92.8% vs. 65/122, 53.3%; *p* < 0.001) ( Table 3 ). Resection with primary anastomosis was significantly more prevalent in UMCG patients compared to HUS patients (36/122, 29.5% vs. 3/69, 4.3%; *p* < 0.001). An open-close procedure occurred more frequently in the UMCG group (17/122, 13.9% vs. 1/69, 1.4%; *p* = 0.004). There were no significant differences in the number of drainage, clip-and-drop or other procedures (in both centers <3%). NEC manifested significantly more frequently in the small intestine in HUS patients compared to UMCG patients (45/69, 65.2% vs. 34/122, 27.9%; *p* < 0.001). Conversely, the colon was significantly more often affected in UMCG patients compared to HUS patients (40/122, 32.8% vs. 6/69, 8.7%; *p* < 0.001). Removal rates of the ileocecal junction were similar between the UMCG (35/122, 38.5%) and HUS (23/69, 33.8%) ( *p* = 0.62). In the UMCG cohort, the median length of small intestine resected was 12 (2–79) cm, and the median length of colon resected was 12 (2–25) cm (missing data *N* = 73). In the HUS cohort, the median length of small intestine resected was 25 (3–80) cm, and the median length of resected colon was 10 (3–25) cm (missing data *N* = 2). We performed a subgroup analysis comparing preoperative clinical characteristics between HUS and UMCG in patients receiving ostomy or primary anastomosis. Patients with ostomy creation had significantly more often deterioration despite maximum conservative treatment as a surgical indication in UMCG (31/65, 47.7% vs. 14/64, 21.9%; *p* = 0.003) and fixed bowel loop in HUS (1/65, 1.5% vs. 13/64, 20.3%; *p* < 0.001). For other surgical indications we found no significant differences between HUS and UMCG in preoperative clinical characteristics including sex, postnatal day of NEC onset or surgery, hours between NEC diagnosis and surgical treatment or preoperative lab values in ostomy creation, or anastomosis patients.

**Table 3:** Intraoperative NEC characteristics

	UMCG ( *n* = 122)	HUS ( *n* = 69)	*P* -value
Drainage procedure preoperatively	2 (1.6)	0 (0.0)	0.54 a
Type of primary procedure performed			
Resection with ostomy creation	65 (53.3)	64 (92.8)	**<0.001** a
Resection with anastomosis	36 (29.5)	3 (4.3)	**<0.001** a
Open-close procedure	17 (13.9)	1 (1.4)	**0.004** a
Clip and drop	1 (0.8)	0 (0.0)	1.00 a
Other	3 (2.5)	1 (1.4)	1.00 a
Location of NEC			
Small intestine	34 (27.9)	45 (65.2)	**<0.001** a
Colonic	40 (32.8)	6 (8.7)	**<0.001** a
Ileocecal	12 (9.8)	6 (8.7)	1.00 a
Multifocal	19 (15.6)	11 (15.9)	1.00 a
Panintestinal	17 (13.9)	1 (1.4)	**0.04** a
Ileocecal junction removed	35 (38.5)	23 (33.8)	0.62 a

Abbreviations: HUS, Helsinki University Hospital; NEC, necrotizing enterocolitis; UMCG, University Medical Center Groningen.

Notes: “Other” included: laparotomy with no resection (due to no necrosis; continued with successful actively conservative treatment) (1), resection without anastomosis (1), and ostomy creation without resection (1).

Data are presented as median (Min.–Max.) or frequency and percentage.

*P* -values of less than 0.05 (two-tailed) were considered statistically significant.

a Fisher's exact test.

### Outcomes at 3 Months

Overall mortality was significantly higher in UMCG patients than HUS (43/122, 35.2% vs. 14/69, 20.3%; *p* = 0.047) ( Table 4 ). When disregarding mortality in patients who underwent an open-close procedure, no statistically significant difference in mortality was observed between the two groups (26/105, 24.8% vs. 13/68, 19.1%; *p* = 0.46). In the UMCG cohort, mortality increased over the 20-year study period (B = 0.130, OR [95% CI] = 1.139 [1.032–1.257], *p* = 0.01) and was associated with reduced gestational age (B = −0.043, OR [95% CI] = 0.958 [0.936–0.979], *p* < 0.001). In the HUS cohort, neither the time period of NEC diagnosis (B = 0.069, OR [95% CI] = 1.072 [0.956–1.201], *p* = 0.23) nor gestational age (B = −0.005, OR [95% CI] = 0.995 [0.971–1.020], *p* = 0.69) was statistically significant predictor of mortality ( Table 5 ). HUS patients received significantly longer postoperative mechanical ventilation (11 [1–88] vs. 4 [0–26]; *p* < 0.001). Post-NEC stenosis rates were similar (14/69, 20.3% vs. 16/122, 13.1%; *p* = 0.22). Recurrent NEC tended to occur more often in the HUS group (7/69, 10.1% vs. 4/122, 3.3%; *p* = 0.06), but overall relaparotomy rates did not differ significantly between the groups (41/122, 33.6% vs. 26/69, 37.7%; *p =* 0.64). The incidence of short bowel syndrome was found to be significantly higher in the HUS group (25/69, 36.2%) in comparison to the UMCG group (18/122, 14.8%) ( *p <* 0.001). Additionally, the total duration of postoperative parenteral nutrition was significantly longer in the HUS group (36 [1–348] vs. 16 [3–80] days; *p <* 0.001).

**Table 4:** Outcomes at 3 months postoperatively

	UMCG ( *n* = 122)	HUS ( *n* = 69)	*P* -value
Overall mortality	43 (35.2)	14 (20.3)	**0.047** a
Mortality excluding open-close procedures	26/105 (24.8)	13/68 (19.1)	0.46 a
Postoperative mechanical ventilation (days) b	4 (0–26)	11 (1–88)	**<0.001** c
Recurrent NEC	4 (3.3)	7 (10.1)	0.06 a
Conservative treatment for recurrent NEC	2 (50.0)	2 (28.6)	0.58 a
Relaparotomy	41 (33.6)	26 (37.7)	0.64 a
Post-NEC stenosis	16 (13.1)	14 (20.3)	0.22 a
Ostomy closure excluding patients deceased before closure	22/48 (45.8)	23/57 (40.4)	0.70 a
Development short bowel syndrome (PN > 60 days postoperatively)	18 (14.8)	25 (36.2)	**<0.001** a
Total postoperative days of parenteral feeding (days) d	16 (3–80)	36 (1–348)	**<0.001** c
Conjugated bilirubin 3 months postoperative (µmol/L)	15 (1–201)	10 (1–242)	0.25

Abbreviations: HUS, Helsinki University Hospital; NEC, necrotizing enterocolitis; PN, parenteral nutrition; total days of parenteral feeding, period from the day following intestinal resection until the final day of parenteral feeding; UMCG, University Medical Center Groningen.

Notes: Data are presented as median (Min.–Max.) or frequency and percentage.

*P* -values of less than 0.05 (two-tailed) were considered statistically significant.

a Fisher's exact test.

b Missing data: 54 UMCG, 6 HUS.

c Mann-Whitney U-Test.

d Missing data: 95 UMCG, 21 HUS.

**Table 5:** Univariable logistic regression analysis of the association between the outcome mortality and the independent variables year of NEC diagnosis and gestational age

Variable	B	OR (95% CI)	*P* -value
HUS			
Year of NEC diagnosis	0.069	1.072 (0.956–1.201)	0.23
Gestational age	−0.005	0.995 (0.971–1.020	0.69
UMCG			
Year of NEC diagnosis	0.130	1.139 (1.032–1.257)	**0.01**
Gestational age	−0.043	0.958 (0.936–0.979)	**<0.001**

Abbreviations: B, unstandardized coefficient; CI, confidence interval; HUS, Helsinki University Hospital; NEC, necrotizing enterocolitis; OR, odds ratio; UMCG, University Medical Center Groningen.

Note: *P* -values of less than 0.05 (two-tailed) were considered statistically significant.

## Discussion

In the current study, we evaluated the clinical presentation, management, and early postoperative outcomes of infants treated surgically for NEC over a 20-year period in two tertiary referral centers in the Netherlands and Finland. We observed a more preterm surgical NEC population in HUS cohort compared to UMCG. The most important findings of the study are that HUS practices lean toward earlier intervention upon NEC diagnosis and more bowel resection with ostomy creation procedures, whereas UMCG is less inclined to do so in this specific study population. In the UMCG, bowel resection with primary anastomosis was performed more often and open-close procedures were almost 10 times more frequent compared to the HUS. Nevertheless, the differences in presentation and management of NEC did not result in a significantly different mortality rate when excluding open-close procedures in this study population.

We found that HUS patients had lower gestational ages and lower birth weights compared to UMCG, which is in line with earlier cohort studies. A previous study in surgically treated infants with NEC in Finland, during 1986–2014, reports a median gestational age of 26 weeks. 4 In a recent Dutch cohort study during 2008–2020, patients with surgical NEC had a median gestational age of 28 weeks, which is comparable to the UMCG infants in our study. 29 The Dutch guidelines for active resuscitation of extremely preterm infants changed in 2006 from 26 + 0/7 to 25 + 0/7 weeks of gestation, and in 2010 to 24 + 0/7 weeks of gestation—which is still the current standard. In Finland, guidelines have allowed active resuscitation of infants from 23 + 0/7 weeks of gestation. This may contribute to the demographic differences in gestational age and birth weight between the two centers and explain the higher prevalence of perforations in the HUS cohort—these tend to occur more in more preterm infants. 30 This could have led to the earlier surgical intervention in the HUS, as pneumoperitoneum is a clear indication for surgical management. It might also be one reason explaining why colonic NEC was seen more often in the UMCG. Several studies reported higher rates of colonic NEC in patients with higher gestational ages, who tend to present earlier in life compared to small intestine NEC 23 31 32 33 —although in association with congenital heart disease, which was an exclusion criterion in our study. In line with these previous findings, UMCG patients had higher gestational ages, but no differences in postnatal day of NEC onset were found between the two centers.

NEC management favors early prevention of deterioration and treatment to control the disease at the “medical NEC” stage, where nonsurgical management suffices, avoiding the risks of surgical intervention in these vulnerable infants. 1 Although the rate of surgical NEC at the UMCG (122/389, 31.4%) was comparable to international figures, a relatively high proportion of surgical NEC cases was observed at the HUS (69/90, 76.7%). 11 12 14 Although a higher ratio of surgical to medically managed NEC cases has been reported in Finland, the reasons for this discrepancy remain unclear. 4 Among the multiple possible indications for surgery in patients with NEC, the presence of pneumoperitoneum is widely considered the most common criterion for surgical intervention. 8 10 34 Conversely, clinical deterioration despite maximum conservative support and a fixed bowel loop for >24 hours are considered relative indications for peritoneal drainage or (exploratory) laparotomy. 10 35 However, the limited sensitivity of pneumoperitoneum as a surgical indication should be noted, as less than half of the infants with intestinal perforation or necrosis exhibit this sign. 8 34 35 Hence, in a common clinical scenario where only relative indications are present, there is room for significant practice variation regarding indications and timing of surgery. The decision to operate on a critically ill infant with NEC may rely largely on the surgeon's training and experience, and on parental preferences. 20 36 37 In both the centers, the attending pediatric surgeon, who is typically involved from Bell's stage IIA onwards, led the process in collaboration with the neonatal team. The final decision for surgery was reached through a multidisciplinary discussion that included the parents, ensuring that all perspectives were considered. It is crucial to acknowledge that the optimal timing of surgery remains unknown with current evidence and multiple indications for surgery can coexist in a given case. 38 Our findings underline the relevance of further exploring this practice variation. The establishment of an international core set of quality indicators for NEC treatment, along with multicenter benchmarking efforts, may allow us to optimize surgical indications, timing, and outcome in NEC. 39

The main operative approach in our surgically treated NEC was laparotomy with resection and ostomy creation. A previous study from Finland found that among 142 infants, the primary procedure performed was intestinal resection with ostomy creation in approximately 70% of patients and resection with anastomosis in around 25%. 4 Conversely, a previous study in the Netherlands showed more modest variations in surgical approaches, revealing that around 45% of cases involved creation of an ostomy, while around 41% of 142 infants with surgical NEC received primary anastomosis. 3 In the present study, we found similar numbers for both surgical approaches in the Dutch UMCG patients, and less primary anastomoses in the Finnish HUS patients than previously reported. Traditionally, ostomy creation is preferred because it avoids the risk of anastomotic leak and stricture associated with primary anastomosis of compromised bowel tissue. 4 8 10 The disadvantages of creation of an ostomy, besides complications of the ostomy itself and the need for a second intervention for closure of the stoma, include increased salt and water loss, risk of a high output stoma, and longer postoperative mechanical ventilation, as observed more frequently in HUS patients. 15 36 40 41 42 Furthermore, HUS patients may have a higher rate of recurrent NEC compared to UMCG, although this difference was not statistically significant in this cohort (7/69, 10.1% vs. 4/122, 3.3%, *p* = 0.06). This finding aligns with a meta-analysis showing that NEC recurrence was more common after resection with ostomy creation compared to resection with primary anastomosis. 15 However, a recent randomized controlled trial comparing anastomosis and ostomy creation for NEC 42 did not find a significant difference in recurrent NEC rates between these approaches. The trial did note that resection with primary anastomosis was associated with a reduced risk of multiple intestinal complications without increasing adverse outcomes. In our study, aside from differences in gestational age and birth weight, there were no clinically relevant preoperative differences between HUS and UMCG patients who received either an ostomy or primary anastomosis. The chosen surgical technique could be attributed to institutional preferences and practice variation. 41 Hopefully, larger datasets and prospective trials can offer guidance in the future.

Previous studies found mortality rates ranging from 30 to 46% in infants who underwent laparotomy due to NEC. 6 13 We observed a total mortality rate of approximately 35% in UMCG and 20% in HUS patients. Only in the UMCG cohort mortality increased over the 20-year study period, potentially linked to the implementation of new Dutch guidelines in 2006 and 2010 prescribing active resuscitation in increasingly extremely preterm infants. 3 As a lower gestational age is a known risk factor for mortality, this may explain the higher mortality rates observed. 43 However, the difference in mortality rates may also be explained by the higher prevalence of panintestinal disease observed in the UMCG patients, which the surgical team deemed incompatible with a reasonable quality of life. Upon excluding these patients with NEC who underwent open-close procedures, the mortality rate of UMCG patients aligned with that of the HUS patients (25% vs. 19%, *p* = 0.46). Panintestinal disease exhibits extensive necrosis that involves almost the entire small and large intestine. Among infants requiring surgery, panintestinal disease occurs in 11% and is associated with almost 100% fatality. 13 44 45 Pathophysiological factors underlying panintestinal NEC remain unclear. It has been established that patients with panintestinal NEC present at older age and less often with pneumoperitoneum, which is in line with our findings. 46

Practice and presentation variation may partially explain the lower occurrence of panintestinal NEC and mortality in HUS patients. The surgical indication of clinical deterioration and a longer time to surgery—as seen more often in UMCG patients—have been associated with higher 28-day postoperative mortality rates. 47 Importantly, ethical and cultural differences between Finland and the Netherlands on the remainder of bowel length considered compatible with a reasonable quality of life after massive intestinal resection may be a significant reason underlying the difference in the occurrence of open-close procedures. Short bowel syndrome, which is often a long-term condition with substantial quality of life implications, 48 49 50 was significantly less common in Dutch patients in our study. Potentially, an end-of-life decision was made in specific Dutch cases of massive bowel necrosis with a high risk of short bowel syndrome, leading to the demise of the child without resection. In contrast, a recent study found that generally only 24% of Nordic patients with pediatric short bowel syndrome remained on parenteral nutrition at 4-year follow-up, 51 possibly prompting toward a decision for resection in such Finnish cases. Additionally, at the HUS, most surgeries were performed in the NICU, which may contribute to explaining the differences in outcomes. Hence, our results may be a partial reflection of the implications attributed to this long-term condition and its quality-of-life prognosis, which may differ between the studied countries.

This study has several strengths. To our knowledge, this is the first European multicenter cohort comparing the presentation and practice variation in surgically treated infants with NEC between two countries over such a long study period. With our data we identified and objectified variations in practice that may guide tailored improvements in care and targeted research efforts, especially in the absence of international evidence-based guidelines on surgical NEC. 52 The results empower international collaboration and benchmarking efforts in tackling this rare disease. Collaborative initiatives such as ERNICA serve as a cornerstone for promoting partnerships between centers of expertise for NEC, enabling the sharing of best practices and expertise across borders. Such collaborations not only provide a platform for consensus-building but also enable large-scale, multicenter research that can strengthen the evidence base for refining clinical guidelines.

Nevertheless, we recognize the limitations of this study. First, for this retrospective analysis, we relied on medical records, which has led to historical data loss in some cases. For the same reason, we only included patients who were intentionally surgically treated for NEC, potentially excluding those unfit for surgery. This also prevented us from including surgery duration, which may be influenced by various procedural factors, complicating its accurate assessment. Second, we did not address specific postoperative surgical complications in our analysis, only the rates of relaparotomy. Most importantly, this study did not investigate long-term outcomes, such as neurodevelopment and bowel function. Further research, preferably in a prospective setup, exploring long-term outcomes in relation to practice variations is recommended. Nevertheless, we find that this study yields valuable insights into practice variation and provides leads for studying the optimization and standardization of NEC care between countries—concerning surgical indication, surgical timing, and surgical technique.

## Conclusion

In this international retrospective cohort of infants treated surgically for NEC, we identified significant practice variation between Finland and the Netherlands. Prime indication for NEC surgery was pneumoperitoneum in HUS cohort and clinical deterioration in UMCG. HUS patients were more often operated within 24 hours, and open-close procedures occurred more frequently in UMCG patients. Excluding open-close procedures, there were no significant differences in mortality rates. We conclude that practice at the UMCG seems to tend more toward a conservative treatment strategy, whereas the HUS seems to be more assertive in initiating surgical management of NEC. Our data highlights the importance of international collaborative initiatives such as ERNICA, focusing on future benchmarking, optimization, and standardization of surgical NEC management.
